# Characterization and Functional Analysis of Pyrabactin Resistance-Like Abscisic Acid Receptor Family in Rice

**DOI:** 10.1186/s12284-015-0061-6

**Published:** 2015-09-11

**Authors:** Xiaojie Tian, Zhenyu Wang, Xiufeng Li, Tianxiao Lv, Huazhao Liu, Lizhi Wang, Hongbin Niu, Qingyun Bu

**Affiliations:** Northeast Institute of Geography and Agroecology, Key Laboratory of Soybean Molecular Design Breeding, Chinese Academy of Sciences, Harbin, 150081 China; Graduate University of Chinese Academy of Sciences, Beijing, 100049 China; Rice Research Institute, Heilongjiang Academy of Land Reclamation Sciences, Jiamusi, 154007 China; Cultivation institute, Heilongjiang Academy of Agricultural Science, Harbin, 150086 China; National Engineering Research Centre for Wheat, Henan Agricultural University, Zhengzhou, 450002 China

**Keywords:** Rice, ABA receptors, PYL, Drought stress, Cold stress, Seed germination

## Abstract

**Background:**

Abscisic acid (ABA) plays crucial roles in regulating plant growth and development, especially in responding to abiotic stress. The pyrabactin resistance-like (PYL) abscisic acid receptor family has been identified and widely characterized in *Arabidopsis*. However, PYL families in rice were largely unknown. In the present study, 10 out of 13 PYL orthologs in rice (*OsPYL*) were isolated and investigated.

**Results:**

Quantitative reverse transcription-polymerase chain reaction (qRT-PCR) analysis showed that expression of *OsPYL* genes is tissue-specific and display differential response to ABA treatment, implying their functional diversity. The interaction between 10 OsPYL members and 5 protein phosphatase 2C in rice (OsPP2C) members was investigated in yeast two-hybrid and tobacco transient expression assays, and an overall interaction map was generated, which was suggestive of the diversity and complexity of ABA-sensing signaling in rice. To study the biological function of *OsPYLs*, two *OsPYL* genes (*OsPYL3* and *OsPYL9*) were overexpressed in rice. Phenotypic analysis of *OsPYL3* and *OsPYL9* transgenic rice showed that *OsPYLs* positively regulated the ABA response during the seed germination. More importantly, the overexpression of *OsPYL3* and *OsPYL9* substantially improved drought and cold stress tolerance in rice.

**Conclusion:**

Taken together, we comprehensively uncovered the properties of *OsPYLs*, which may be good candidates for the improvement of abiotic stress tolerance in rice.

**Electronic supplementary material:**

The online version of this article (doi:10.1186/s12284-015-0061-6) contains supplementary material, which is available to authorized users.

## Background

Abscisic acid (ABA) plays pivotal roles in regulating plant growth and development, including seed dormancy, germination, and seedling growth. More importantly, ABA is the key phytohormone that functions in a plant’s response to abiotic stressors such as drought, high salinity, and extreme temperature (Cutler et al. 2010). During abiotic stress, ABA biosynthesis is activated, resulting in an increase in ABA levels in the plant. ABA binds to the pyrabactin resistant-like/regulatory components of ABA receptors, PYL/RCAR (hereafter referred to as PYLs for simplicity), an ABA receptor family that promotes the interaction between PYL with protein phosphatase 2C (PP2C), which then results in the release of SNF1-related protein kinase (SnRK)) from the repression of PP2C. Finally, the active SnRK phosphorylates and activates downstream transcriptional factors that promote the expression of ABA-regulated genes. An ABA signal is then generated, which in turn results in the acquisition of abiotic stress resistance in plants (Ma et al. 2009; Park et al. 2009; Cutler et al. 2010; Klingler et al. 2010). ABA receptor PYL proteins, which contain a conserved steroidogenic acute regulatory-related lipid transfer (START) protein domain, are the core components of this ABA sensing signaling pathway (Ma et al. 2009; Park et al. 2009). To date, PYL proteins have been identified from distinct plant species, including *Arabidopsis*, rice, tomato, and soybean, which present highly conserved PYL-mediated ABA-sensing signaling pathways (Ma et al. 2009; Park et al. 2009; Kim et al. 2012; Bai et al. 2013; Gonzalez-Guzman et al. 2014; He et al. 2014). The biochemical property and structure of PYLs have been extensively studied in the dicot plant model *Arabidopsis* (Ma et al. 2009; Melcher et al. 2009; Klingler et al. 2010; Joshi-Saha et al. 2011). Some AtPYLs are monomers that facilitate interactions with PP2C in the absence of ABA; some AtPYLs are in a dimeric state and require ABA to form a complex with PP2C (Hao et al. 2011). Recent studies have investigated the biological functions of various *AtPYLs*. The overexpression of *AtPYL5* leads to ABA hypersensitivity during early seedling development, as well as enhanced drought stress tolerance (Klingler et al. 2010). *AtPYL8* is involved in root growth and development, which is in line with its root-specific expression pattern (Antoni et al. 2013). Although AtPYL13 is not an ABA receptor, it can positively regulate the ABA signaling pathway by interacting with and inhibiting both the PYL receptors and the PP2C co-receptors (Li et al. 2013; Zhao et al. 2013). AtPYL4^A194T^ forms stable complexes with PP2CA in the absence of ABA, and the overexpression of AtPYL4^A194T^ increases a plant’s sensitivity to ABA-mediated inhibition of germination and seedling establishment, as well as enhances drought resistance (Pizzio et al. 2013). In addition, AtPYLs are largely functionally conserved, and the analysis of higher order mutants have indicated that AtPYLs regulate stomatal conductance (Gonzalez-Guzman et al. 2012).

Unlike AtPYLs, information on PYL homologs in rice is limited. Studies have predicted that rice has 13 OsPYL members that share high sequence similarity with AtPYLs (Kim et al. 2012; He et al. 2014). Core components in ABA signaling, including OsPYLs, OsPP2C, OsSAPK2, and OsOREB1, have been identified in rice, and the ABA signaling transduction pathway has also been reconstituted in a protoplast system (Kim et al. 2012). The biochemical properties and structure of OsPYLs have also been recently reported (He et al. 2014). However, to date, only OsPYL/RCAR5 has been identified as a positive regulator in seed germination, early seedling growth, and drought and salt stress tolerance (Kim et al. 2012; Kim et al. 2014). Other features of OsPYLs such as expression pattern, subcellular localization, interaction specificity with OsPP2C, and biological function, have not been examined.

The present study examined the tissue-specific expression pattern and distinct response of *OsPYL* members to ABA treatment, which was suggestive of its differential biological function. An overall interaction map between 10 OsPYLs and 5 OsPP2C members indicated that OsPYLs were selective of their interaction partner, thus indicating the complexity and specificity of the rice ABA-sensing signaling pathway. Furthermore, using *OsPYL3* and *OsPYL9*, we determined that *OsPYLs* play pivotal roles in ABA-mediated inhibition of seed germination, as well as drought and cold stress tolerance, which thereby could serve as potential targets for the improvement of abiotic stress tolerance in rice.

## Results

### Expression Pattern of *OsPYL* Members

**Fig. 1:**
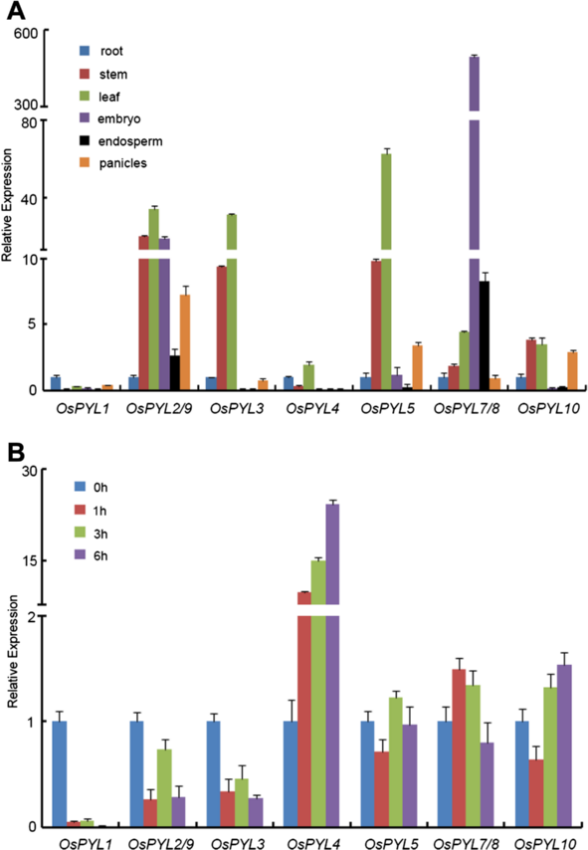
Tissue-specific and ABA-regulated expression of *OsPYLs* genes. **a** Tissue-specific expression of *OsPYLs* was analyzed by quantitative RT-PCR in roots, leaves, stems, embryo, endosperm and panicles. The expression level of *OsPYLs* in roots was set as 1 and the fold change was analyzed via the 2^-△△CT^ method using the rice *ubiquitin* gene as an internal control. Values represent the mean ± SD of three biological replicates. **b** ABA-regulated expression of *OsPYLs* was analyzed by quantitative RT-PCR. Two-week-old seedlings were incubated in liquid MS medium containing 200 μM ABA for 0, 1, 3 and 6 h. The expression level of *OsPYLs* at 0 h was set as 1 and the fold change was analyzed via the 2^-△△CT^ method using the rice *ubiquitin* gene as an internal control. Values represent the mean ± SD of three biological replicates

Using the *AtPYLs* sequence as queries and search in the rice genome database, a total of 13 *OsPYL* orthologs were identified in rice. Among these, three (*OsPYL*11–13) are thought to be nonfunctional that were caused by a large fragment deletion in the N or C terminal of the gene (Kim et al. 2012). To obtain an overview of the expression pattern of *OsPYL* members in different tissues, quantitative RT-PCR was performed. Because of the high level of sequence similarity among *OsPYL* members, it was difficult to design primers that could discriminate between *OsPYL2* and *OsPYL9*, and *OsPYL7* and *OsPYL8*. Most of the *OsPYLs* were detected in all tissues, although differentially expressed (Fig. 1a). *OsPYL7*/*8* was upregulated in embryos, *OsPYL3* and *OsPYL5* were upregulated in leaves, *OsPYL1* in roots, and *OsPYL2*/*9* in all tissues (Fig. 1a). These diverse tissue-specific patterns of *OsPYLs* were indicative of its diverse biological functions.

*OsPYLs* are also differentially expressed after ABA treatment (Fig. 1b). Some *OsPYLs* were downregulated such as *OsPYL1*, *OsPYL2*/*9*, and *OsPYL3* (Fig. 1b), whereas *OsPYL4* was upregulated (Fig. 1b). The expression of *OsPYL5*, *OsPYL7*/*8*, and *OsPYL10* was not affected by ABA treatment (Fig. 1b). These findings suggest that *OsPYL* members play diverse roles in sensing the ABA signal.

### OsPYLs are Localized in the Cytosol and Nucleus

**Fig. 2:**
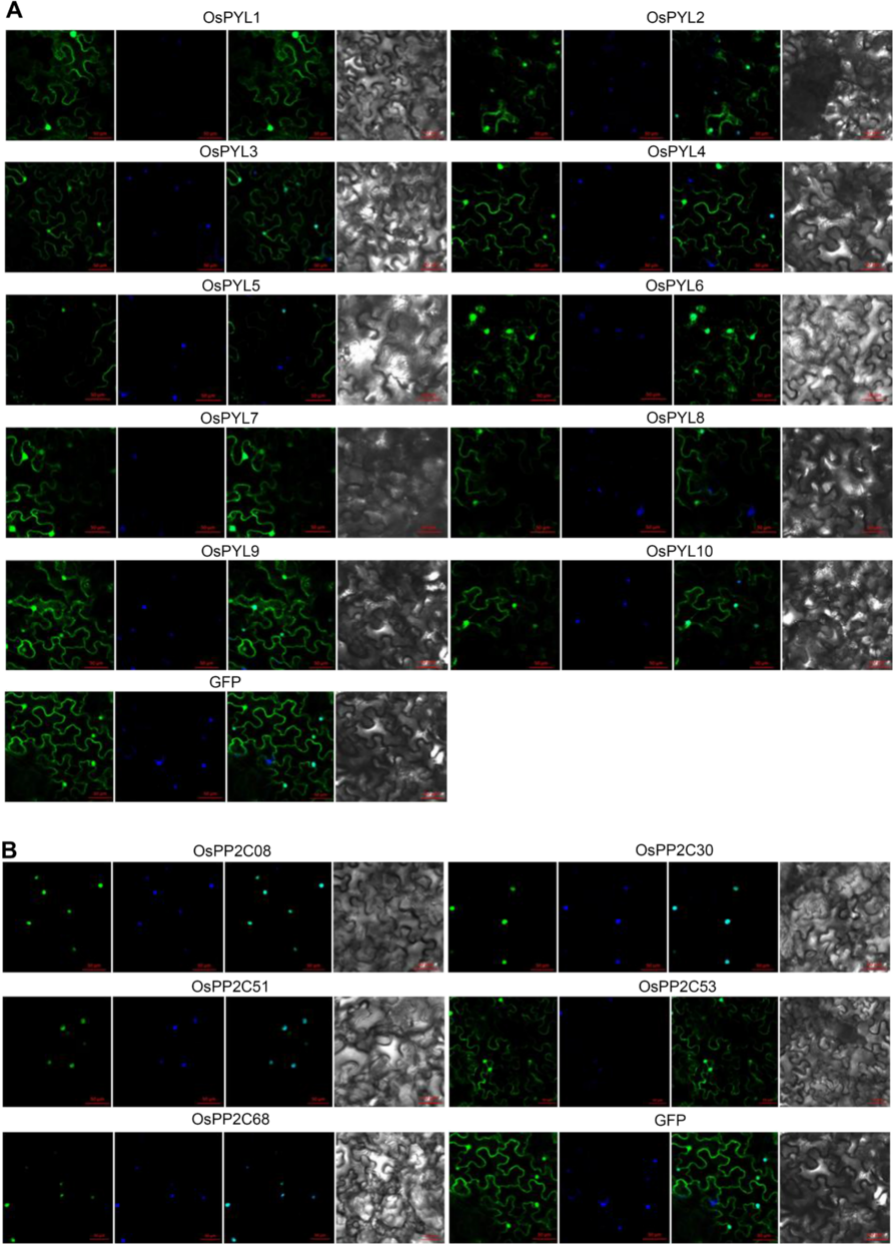
Subcellular localization of OsPYLs and OsPP2Cs. Confocal images were taken from Nicotiana benthamiana leaves epidermal cells. Constructs of (GFP)-OsPYLs (**a**) and (GFP)-OsPP2Cs (**b**) driven by the 35S promoter were infiltrated and observed at 3 days later. From left panel to right panel are GFP image, DAPI dye image, merged image and bright-field image. Empty GFP vector was used as control. The positions of nuclei were shown by DAPI staining

To determine the subcellular localization of OsPYLs, green fluorescent protein (GFP)-OsPYL fusion proteins driven by a 35S promoter were transiently expressed in *Nicotiana benthamiana* leaves. All tested OsPYLs were localized in both the cytosol and nucleus (Fig. 2a), which were consistent with the results involving AtPYL9 and soybean GmPYL members (Ma et al. 2009; Bai et al. 2013). These results indicated that PYLs of different plant species are localized in the same cellular regions, which might partly explain the functional conservation of PYL proteins as ABA receptors.

### OsPYL Members Selectively Interact with OsPP2C Members

**Fig. 3:**
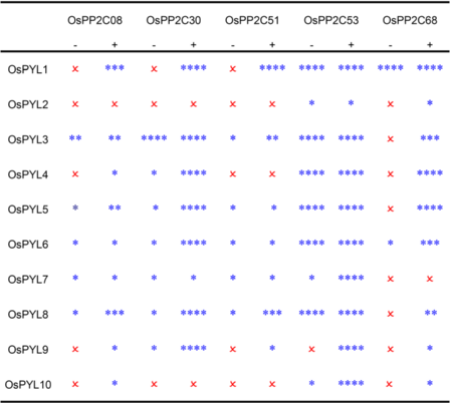
Interaction map between OsPYLs and OsPP2Cs. Interaction between OsPYLs and OsPP2Cs was first examined in yeast two-hybrid, “–” and “+” indicated without and with ABA supplement. Number of “*” indicate the interaction strength from weak to strong, one (*) to four star (****) represent that the yeast colony can grow on SD/-His/-Leu/-Trp plate containing different concentration of 3-AT (0, 3.0, 5.0 and 10 mM). “x” means no interaction. These interactions were further confirmed by BiFC in tobacco transient expression system

**Fig. 4:**
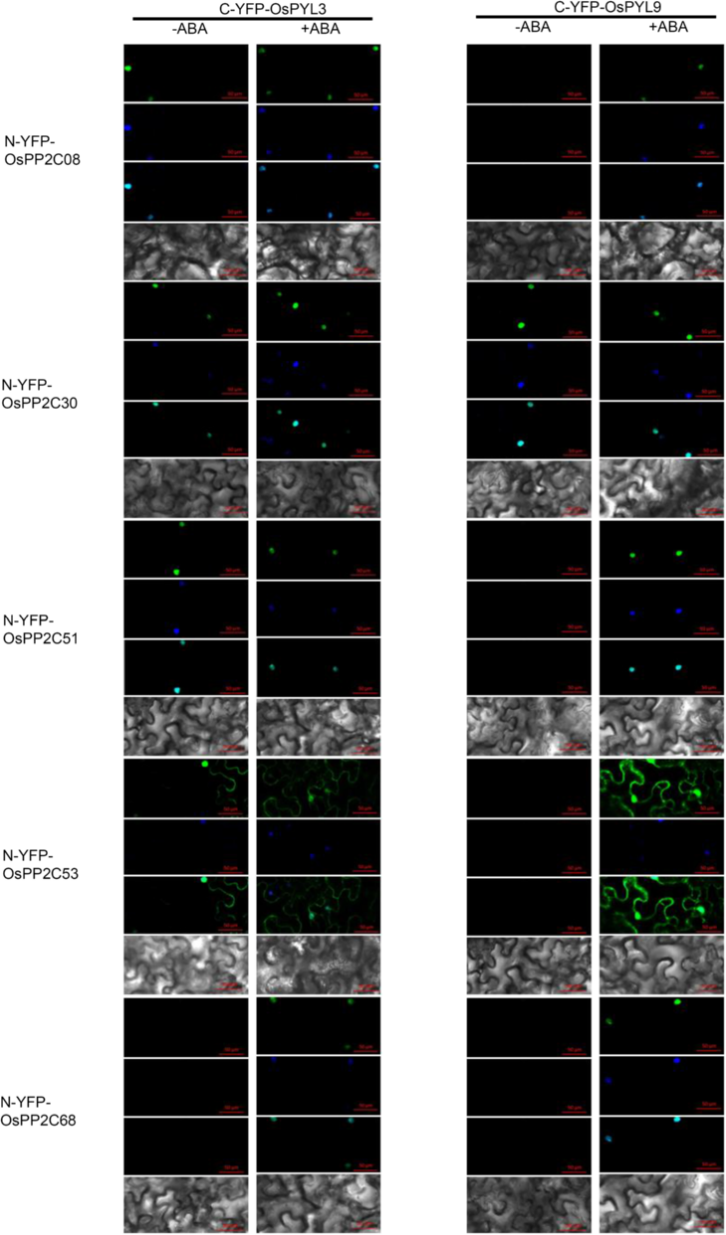
OsPYL3 and OsPYL9 interact with OsPP2Cs in *planta*. In *planta* interaction and subcellular localization analysis using agroninfiltrated *Nicotiana benthamiana* leaves. The interaction was detected by fluorescence in BiFC analysis. 100 μM ABA was injected at 24 h before observation. From upper panel to bottom panel are YFP image, DAPI dye image, merged image and bright-field image. The positions of nuclei were shown by DAPI staining

PYL family proteins, as functional ABA receptors, interact with clade A PP2Cs to form PYL-ABA-PP2C triple complexes that facilitate in the transmission of ABA signals (Cutler et al. 2010; Joshi-Saha et al. 2011). A total of 10 predicted clade A OsPP2Cs have been identified in rice (Xue et al. 2008). In the present study, five clade A OsPP2Cs were isolated and investigated (Fig. 2b). To determine whether OsPYLs interacted with these OsPP2Cs, yeast two-hybrid assays were conducted in the absence or presence of ABA. The results showed that the interaction between OsPYLs and OsPP2Cs was selective and specific (Fig. 3, Additional file 1: Figure S1). Some OsPYLs interacted with all OsPP2C members, except for OsPYL2 and OsPYL10. Most interactions were ABA-dependent or ABA-enhanced. A few interactions were ABA-independent or constitutive such as that observed with OsPYL1 and OsPP2C53 (Fig. 3). Most OsPYLs strongly interacted with OsPP2C30 and OsPP2C53 in an ABA-dependent or ABA-independent manner, respectively (Fig. 3). These results demonstrated that OsPYLs bind to OsPP2Cs in diverse fashions and with different intensities. To confirm these interactions in plant cells, BiFC experiments in *N. benthamiana* leaves were performed. The *in vivo* interaction results indicated that these could reproduce, as well as validated the results of the yeast two-hybrid assay (Fig. 4). Based on the results of the yeast two-hybrid and BiFC assays, an overall interaction map was generated between the 10 OsPYLs and 5 OsPP2C members (Fig. 3). These results provide evidence that the tested OsPYLs were capable of functioning as ABA receptors, and that the ABA-sensing mechanism was conserved among different plants.

### OsPP2C Determines the Subcellular Localization of the OsPYLs-OsPP2C Complex in an ABA-independent Manner

The results of the BiFC assay indicated that the subcellular localization of the fused green fluorescent proteins varied among different OsPYLs and OsPP2C members, and most of the OsPYL-OsPP2C complexes were localized in the nucleus (Fig. 4), which is not consistent with the localization of OsPYLs (Fig. 2a). To explore the underlying mechanism of this activity, the subcellular localization of five OsPP2C members was investigated. OsPP2C53 was localized to both the nucleus and cytosol, whereas other OsPP2C members were detected only in the nucleus (Fig. 2b). Comparative analysis showed a similarity between the subcellular localization of OsPP2C members and that of OsPYLs-OsPP2C complexes (Figs. 2b and 4). In the BiFC assay, ABA was also injected to test whether ABA affected the interaction between OsPYLs and OsPP2Cs. The results showed that ABA treatment only enhanced interaction strength, and did not affect the subcellular localization of the OsPYL-OsPP2C complexes (Fig. 4). Consequently, the findings indicated that OsPP2C members can determine the subcellular localization of OsPYL-OsPP2C complexes in an ABA-independent manner.

### Overexpression of *OsPYL3* and *OsPYL9* Confers ABA Hypersensitivity during Seed Germination

**Fig. 5:**
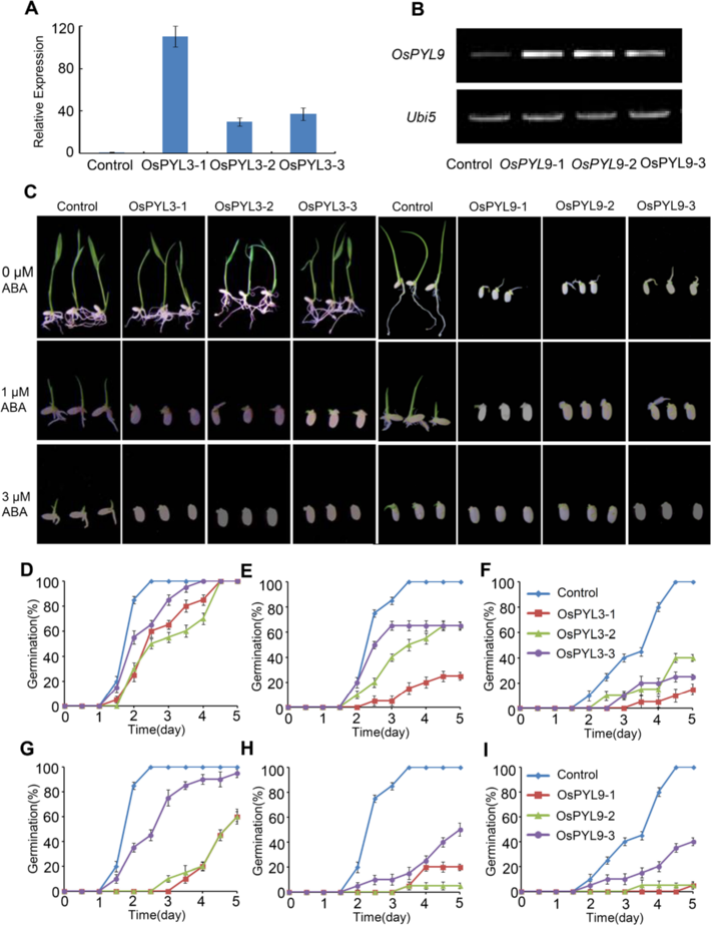
Overexpression of *OsPYL3* and *OsPYL9* confer ABA hypersensitivity during seed germination. **a** Quantitative RT-PCR analysis of *OsPYL3* in overexpression transgenic lines. The expression level of *OsPYL3* in control line was set as 1 and the fold change was analyzed via the 2^-△△CT^ method using the rice *ubiquitin* gene as an internal control. Values represent the mean ± SD of three biological replicates. **b** RT-PCR analysis of *OsPYL9* in overexpression transgenic lines. Full length *OsPYL9* primer was used and rice *ubiquitin* transcripts were used as control. **c** Representative photographs of seed germination of *OsPYL3* and *OsPYL9* overexpression transgenic lines. Seeds of control and transgenic lines were grown on half-strength MS medium containing indicated concentration of ABA for 5 days. Photographs were taken on day 5. (D to I) Germination time course of *OsPYL3* (**d**-**f**) and *OsPYL9* (**g**-**i**) overexpression transgenic lines and control in medium without ABA (**d** and **g**), 1 μM ABA (E and H) and 3 μM ABA (**f** and **i**). Data show the mean ± SD of three replicates. At least 50 seeds per genotype were measured in each replicate

To determine whether *OsPYLs* are functional ABA receptors in rice, *OsPYL3* and *OsPYL9* were selected for further investigation. Constructs of *OsPYL3* and *OsPYL9* driven by the *35S* promoter were transformed into rice, and more than 20 independent transgenic lines were produced for each gene. Three independent transgenic lines of each gene were chosen for further analysis. The expression levels of *OsPYL3* and *OsPYL9* in the transgenic lines were measured by quantitative RT-PCR and RT-PCR, respectively (Fig. 5a and b). *OsPYL3* and *OsPYL9* were overexpressed in the transgenic lines compared to the control lines (Fig. 5a and b). A seed germination assay was conducted to examine the ABA-related phenotype of *OsPYL3* and *OsPYL9* overexpression lines. In the absence of ABA, *OsPYL3* and *OsPYL9* overexpression lines showed a slight delay in germination rate, and the *OsPYL9* overexpression line germinated at a slower rate compared to that of the *OsPYL3* overexpression line (Fig. 5c, d, and g). Application of different concentrations of ABA showed that the seed germination rate of the *OsPYL3* and *OsPYL9* overexpression lines was markedly slower than that of the controls (Fig. 5c, e, f, h, and i). *OsPYL9* overexpression lines hardly germinated in a medium containing 3 μM of ABA until 5 days later (Fig. 5i). This finding indicated that the overexpression of *OsPYL3* and *OsPYL9* conferred ABA-hypersensitivity during seed germination and functioned as an active ABA receptor. In addition, a higher expression level of *OsPYL9* than that observed with *OsPYL3* in embryos may explain its enhanced phenotype during ABA-regulated seed germination (Figs. 1a and 5).

### Overexpression of *OsPYL3* and *OsPYL9* Enhances Drought Stress Tolerance

**Fig. 6:**
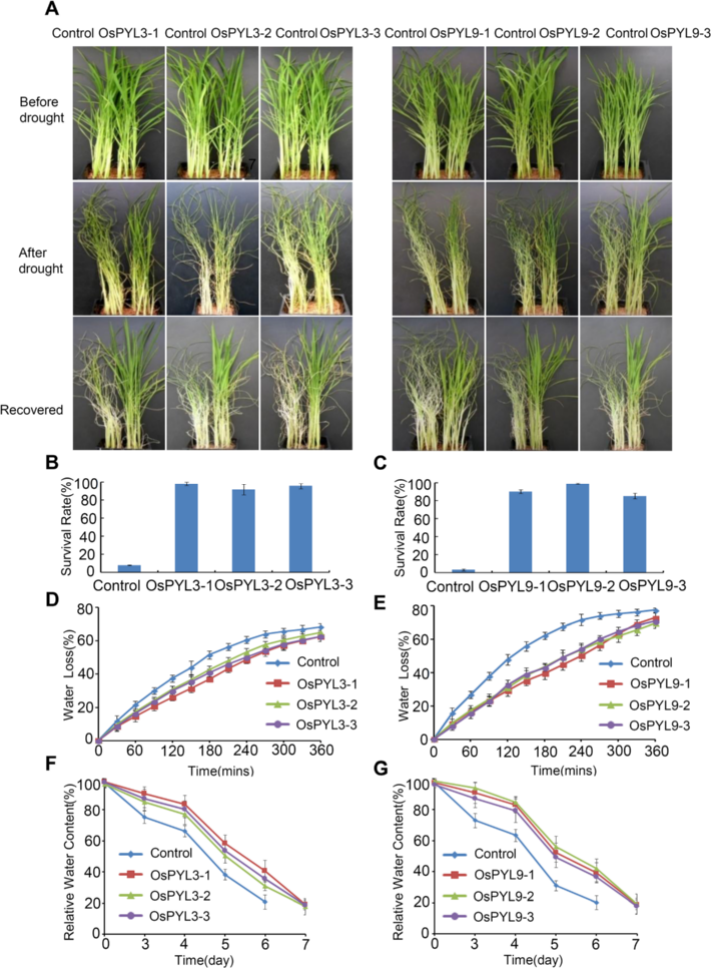
Overexpression of *OsPYL3* and *OsPYL9* exhibit enhanced drought stress tolerance. **a** Three-week-old *OsPYL3* and *OsPYL9* overexpression seedlings were subjected to drought conditions by withholding water and then rewatered. Photographs were taken before drought (upper panel) and 10 dayays after drought (middle panel), and 7 days after rewatering (bottom panel). **b** and **c** Survival rates of the drought treated *OsPYL3* (B) and *OsPYL9* (C) overexpression seedlings after 7 days of rewatering. Values are mean ± SD (*n* = 30 for each replicate) of three independent experiments. **d** and **e** Water loss rate of *OsPYL3* **d** and *OsPYL9* (**e**) overexpression lines. Leaves of the same developmental stages were excised and weighed at various time points after detachment. Values are means ± SD of three individual plants per genotype. Experiments were repeated at least three times with similar results. (**f** and **g**) Relative water content of *OsPYL3* (**f**) and *OsPYL9* (**g**) overexpression lines. Seedling of similar stage was withhold water for indicated days and used for measure relative water content. Values are means ± SD of three individual plants per genotype. Experiments were repeated three times

To further investigate the biological function of *OsPYLs*, *OsPYL3* and *OsPYL9* overexpression lines were subjected to a drought tolerance assay. Three-week-old seedlings of similar sizes showed the capability of withholding water for 10 days, control seedling leaves were curved and wilted and its seedling had fallen down, whereas *OsPYL3* and *OsPYL9* overexpression lines remained upright (Fig. 6a). After 7 days of re-watering, over 80 % of the *OsPYL3* and *OsPYL9* overexpression lines were turgid and survived; in contrast, only less than 10 % of the control lines remained alive (Fig. 6a–c). These results indicated that the ectopic expression of *OsPYL3* and *OsPYL9* enhanced drought stress tolerance in rice (Fig. 6a–c).

Plants lose water mainly through stomata transpiration. To determine whether stomatal closure is involved in the enhanced drought tolerance of *OsPYL3* and *OsPYL9* overexpression lines, a water-loss assay was performed using detached leaves. The water loss rate of the *OsPYL3* and *OsPYL9* overexpression lines was significantly slower than that observed in the controls (Fig. 6d and e). Relative water content (RWC) more accurately reflects the physiological consequence of cellular water deficit. Fig. 6e and g shows that with drought stress, the *OsPYL* overexpression lines lost water at a slower rate and have a lower minimum relative water content that helped the plants survive during severe drought conditions. These results indicated that the *OsPYL3* and *OsPYL9* overexpression lines have a faster rate of stomatal closure and were hypersensitive to drought treatment compared to the controls, thereby contributing to a high level of drought stress tolerance.

### Overexpression of *OsPYL3* and *OsPYL9* Increases Cold Stress Tolerance

**Fig. 7:**
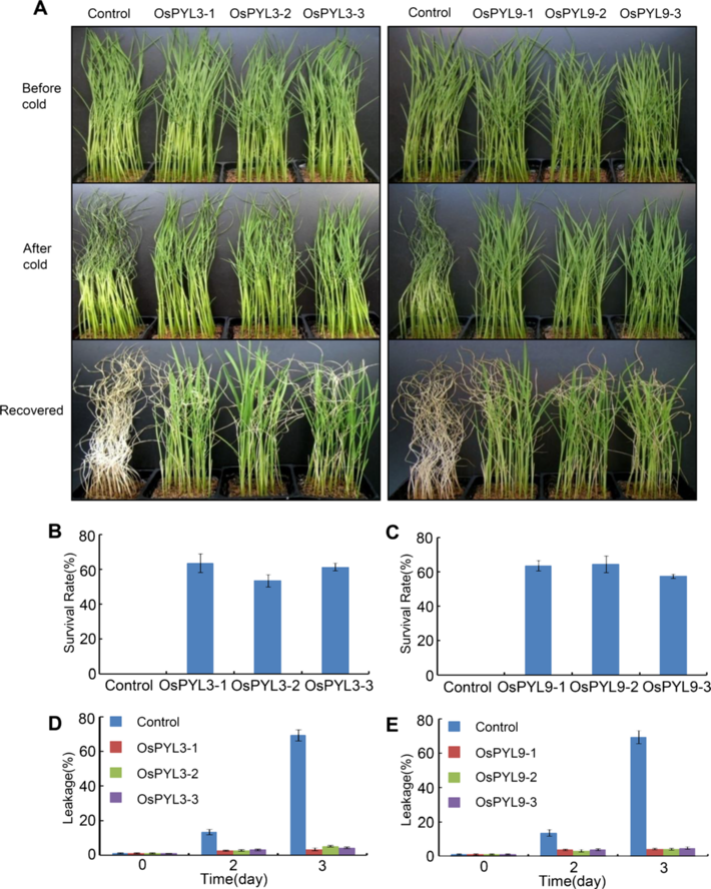
Overexpression of *OsPYL3* and *OsPYL9* enhanced cold stress tolerance. **a** Two-week-old *OsPYL3* and *OsPYL9* overexpression seedlings were subjected to 10 °C treatment and then recovered at room temperature. Photographs were taken at before cold (upper panel) and 4 days after cold treatment (middle panel), and 7 days after recovered (bottom panel). **b** and **c** Survival rates of the cold-treated plants after 7 days of recovery. Values are mean ± SD (*n* = 30 for each replicate) of three independent experiments. **d** and **e** Relative ion leakage in rice leaves after 10 °C treatment for 0 d, 2 d and 3 d. Data represent means ± SD of three biological replicates

The rice seedlings were also subjected to cold stress. To examine whether *OsPYLs* played a role during cold stress response, a cold tolerance assay was performed. Two-week-old rice seedlings of similar size were incubated in a 10 °C growth chamber for 4 days. After 4 days, the leaves of the transgenic lines remained green and flats, and only the leaf tips were rolled up. In contrast, the leaves of the control lines were wilted, rolled up, and dry (Fig. 7a). After 7 days of recovery at the normal temperature, the survival rate of the *OsPYL3* and *OsPYL9* transgenic plant was >50 %, compared to the >95 % death rate of the control lines (Fig. 7a-c). To evaluate the effect of cold stress on cell membranes, two-week-old rice seedlings were exposed to a 10 °C environment, and relative ion leakage was measured. Without cold treatment, the relative ion leakage rate of the transgenic lines and control lines was similar. After 3 days cold treatment, the relative ion leakage rate of the control lines was 70 %, whereas that of the *OsPYL3* and *OsPYL9* overexpression lines was <10 % (Fig. 7d and e). These findings indicated that the increased cell membrane stability of *OsPYL3* and *OsPYL9* overexpression lines during cold stress partially, if not totally, contributed to the observed enhanced tolerance of cold stress. These results indicated that overexpression of *OsPYL3* and *OsPYL9* substantially improved cold stress tolerance in rice.

### Expression of ABA-Regulated Genes is Enhanced in *OsPYL3* and *OsPYL9* Overexpression lines

**Fig. 8:**
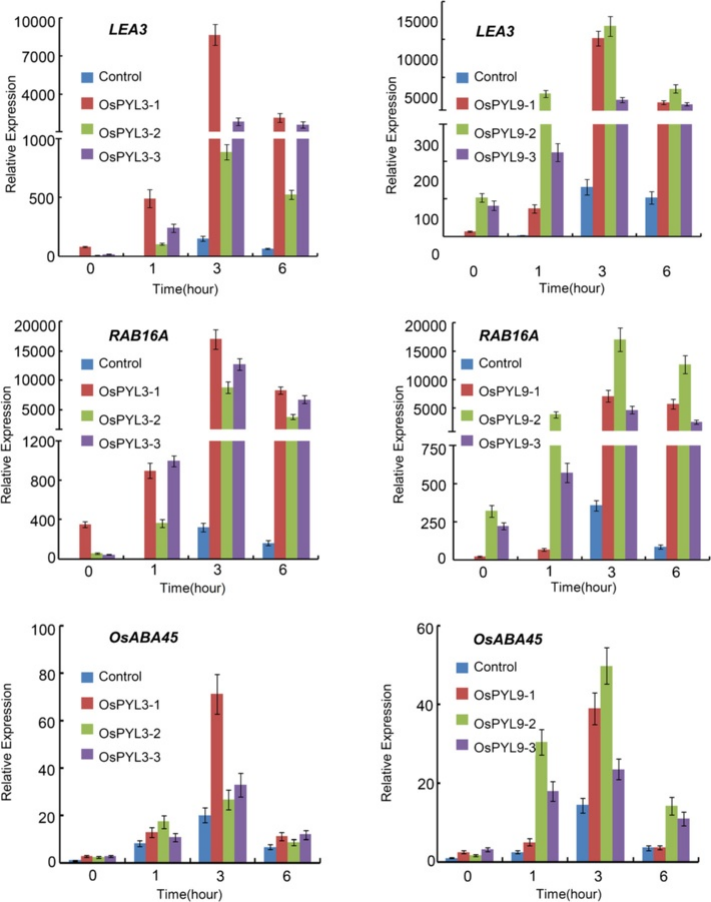
Overexpression of *OsPYL3* and *OsPYL9* increased the expression of ABA responsive genes. Fourteen-day-old control, *OsPYL3* and *OsPYL9* overexpression seedlings were treated with ABA solution (100 μM) for indicated time. Shoots were analyzed by quantitative RT-PCR. *LEA3* (*LOC*_*Os05g46480*), *RAB16A* (*LOC*_*Os11g26790*), and *OsABA45* (*LOC*_*Os12g29400*) were monitored. The expression level of ABA regulated gene in control at 0 h was set as 1 and the fold change was analyzed via the 2^-△△CT^ method using the rice *ubiquitin* gene as an internal control. Values represent the mean ± SD of three biological replicates

Because *OsPYL3* and *OsPYL9* overexpression lines showed significant drought and cold stress tolerance at the seedling stage, we were prompted to determine whether the expression of ABA-regulated genes was also enhanced in these transgenic lines. Several ABA-regulated genes (*LEA3*, *RAB16A*, and *OsABA45*) were selected, and its expressions levels between the control and *OsPYL3* and *OsPYL9* overexpression lines in response to ABA treatment were compared. Quantitative RT-PCR analysis indicated that ABA induced the expression of these genes, which was significantly higher than that observed in the controls (Fig. 8). Notably, in the absence of ABA treatment, the expression of these ABA-regulated genes was still higher in the *OsPYL3* and *OsPYL9* overexpression lines than that in the control, indicating that the overexpression of *OsPYL3* and *OsPYL9* promoted the constitutive expression of ABA-regulated genes (Fig. 8), which possibly explains the delayed seed germination of *OsPYL3* and *OsPYL9* overexpression lines in the absence of ABA (Fig. 5c, d, and g). Taken together, the upregulation of ABA-regulated genes might also have contributed to the observed ABA hypersensitivity and the enhanced stress tolerance of the *OsPYL3* and *OsPYL9* overexpression lines.

## Discussion

Members of the PYL protein family such as the ABA receptors and the core component in ABA-sensing signaling have been investigated in various plant species, including *Arabidopsis*, soybean, rice, and cucumber (Ma et al. 2009; Wang et al. 2012; Bai et al. 2013; He et al. 2014; Kim et al. 2014). Compared to the extensive studies on AtPYLs, the property, function, and mechanism of OsPYLs are largely unknown. In the present study, based on amino acid sequence analysis of AtPYLs and predicted OsPYLs in the rice genome annotation project, 10 out of the 13 *OsPYLs* were cloned and studied. *OsPYLs* were differentially expressed in the leaves, stems, roots, panicles, embryo, and endosperm, and some *OsPYL* members showed pronounced tissue-specific expression patterns (Fig. 1a). *OsPYLs* also displayed a differential response to ABA treatment (Fig. 1b). The diverse expression patterns of *OsPYLs* were indicative of their functional diversity. All OsPYLs were localized to the cytoplasm and nucleus (Fig. 2a), which was consistent with the results of previous reports on the subcellular localization of AtPYLs and GmPYLs (Ma et al. 2009; Bai et al. 2013).

The interaction between the OsPYLs and OsPP2Cs was investigated in both yeast and tobacco, and an overall interaction map was generated between 10 OsPYLs and 5 OsPP2C members in the absence or presence of ABA treatment (Figs. 3 and 4). OsPYLs selectively interacted with OsPP2Cs in ABA-dependent or ABA-independent manner. Combined with a recent report that OsPYLs inhibit OsPP2C activity (He et al. 2014), these data suggest that OsPYLs are functional rice ABA receptors.

In addition to the discovery of PYLs as ABA receptors, the present study has shown that these proteins also regulate ABA signaling and improve tolerance to abiotic stress. Overexpression of *AtPYL4* and *AtPYL5* leads to enhanced ABA hypersensitivity during seed germination and improved drought tolerance (Santiago et al. 2009; Pizzio et al. 2013). In addition, the root-specific expression of *AtPYL8* regulates ABA-mediated inhibition of root growth (Antoni et al. 2013). However, information on the biological function of *OsPYLs* in rice, which is considered a monocot model, is limited. *OsPYL5* has been shown to positively regulate seed germination and drought stress response (Kim et al. 2012; Kim et al. 2014). In the present study, transgenic rice overexpressing *OsPYL3* and *OsPYL9* were produced and analyzed in detail (Fig. 5a and b). First, overexpression of *OsPYL3* and *OsPYL9* conferred ABA hypersensitivity during seed germination (Figs. 5c-i). In addition, overexpression of *OsPYL3* and *OsPYL9* in the absence of ABA significantly delayed seed germination, and *OsPYL9* played more important roles, which was consistent with the upregulation of *OsPYL9* in the embryo compared to that of *OsPYL9* (Fig. 5c-i). Second, overexpression of *OsPYL3* and *OsPYL9* enhanced drought stress tolerance, which can be partly explained by a slower water loss rate through the stomata in transgenic lines (Fig. 6). Third, *OsPYL3* and *OsPYL9* overexpression lines show increased membrane stability during cold stress and enhanced cold stress tolerance (Fig. 7). Last, expression of ABA-regulated genes in *OsPYL3* and *OsPYL9* overexpression lines was significantly higher than that in the controls (Fig. 8), which accounted for the increased stress tolerance of transgenic plants. Interestingly, compared to the controls, *OsPYL3* and *OsPYL9* overexpression lines did not show any negative effect on growth, grain yield, and other observable phenotypes, whereas these were observed in *OsPYL5* (Kim et al. 2014). One possible explanation for this finding is that *OsPYL5* was directed by the maize *ubiquitin* promoter, which is a very strong promoter in monocots, and *OsPYL3* and *OsPYL9* was directed by the *35S* promoter, whose efficiency was less than that of *ubiquitin* in rice. Taken together, these results indicate that *OsPYL3* and *OsPYL9* can be used as a target gene for the improvement of abiotic stress tolerance in rice.

## Conclusions

In the present study, we study the expression pattern of PYL orthologs in rice (OsPYL), generate an overall interaction map between 10 OsPYL members and 5 protein phosphatase 2C in rice (OsPP2C) members and show the biological function ofOsPYL3 and OsPYL9. Taken together, we comprehensively uncovered the properties of OsPYLs, which may be good candidates for the improvement of abiotic stress tolerance in rice.

## Methods

### Plant materials, and rice transformation

For cloning of *OsPYLs* and *OsPP2Cs* genes, *Oryza sativa* L. ssp. *Japonica* cv. Nipponbare was used. The coding sequence of *OsPYLs* and *OsPP2Cs* were cloned from the cDNA using standard PCR-based protocol. The primers used are listed at Additional file 1: Table S1. Full-length sequences of *OsPYLs* and *OsPP2Cs* were cloned into pENTR/D-Topo (Invitrogen), resultant constructs were confirmed by sequencing and saved for later use. To over express the *OsPYLs* in rice, *OsPYLs* was transferred to *pH7WG2* vector via LR recombination reaction of the Gateway system. The resultant *35S:OsPYLs* constructs, in which *OsPYLs* was drived by the cauliflower mosaic virus (CaMV) *35S* promoter, were transferred into *Agrobacterium tumefaciens* EHA105. Finally, *OsPYLs* were transformed into *Oryza sativa* L. ssp. *Japonica* cv. Longjing 11 by the *Agrobacterium*-mediated co-cultivation method.

### Yeast two-hybrid assay

Full-length sequences of *OsPYLs* and *OsPP2Cs* were cloned into *pDEST32* or *pDEST22* vector via an LR recombination reaction and used as baits or preys respectively. The resultant constructs were transformed into the yeast strain Y2H gold. Presence of the transgenes was confirmed by growth on a SD/-Leu/-Trp plate. To assess protein interactions, the transformed yeast cells were suspended in liquid SD/-Leu/-Trp to OD_600_ = 1.0. The suspended cells were spread on plates containing SD/-His/-Leu/-Trp medium supplied with indicated concentration of 3-AT (3-amino-1, 2, 4-triazole) and ABA. The interactions were observed after 4 days of incubation at 30 °C. The experiments were repeated three times with similar results.

### Subcellular localization and Bimolecular fluorescence complementation in tobacco

For subcellular localization of OsPYLs and OsPP2Cs, full-length sequences of OsPYLs and OsPP2Cs were cloned into the pH7WGF vector via an LR recombination reaction, in which the OsPYLs and OsPP2Cs fused with green fluorescent protein (GFP) was drived by 35S promoter. For Bimolecular fluorescence complementation (BiFC) assays, OsPYLs were fused with the C-terminal portion of the yellow fluorescent protein (YFP) via an LR recombination reaction; OsPP2Cs were fused with the N-terminal portion of the yellow fluorescent protein (YFP) via an LR recombination reaction. For transient expression, the fusion constructs were transferred into *Agrobacterium tumefaciens* GV3101. *Agrobacterium tumefaciens* strain harboring each construct along with the *p19* strain were infiltrated into 4-week-old *N. benthamiana* leaves. For staining of the nuclei, 10 mg/ml 4, 6-diamidino-2-phenylindole (DAPI) was infiltrated into *N. benthamiana* leaves 3 h before observation. For microscopic analyses, leaf discs were cut 3 d after infiltration. The fluorescence signal was observed using confocal microscopy.

### Analysis of genes expression

Total RNA was extracted using TRIzol (Invitrogen) and treated with DNaseI. cDNA was synthesized from 2 µg of total RNA using SuperscriptII Reverse Transcriptase. Real-time PCR was performed with SYBR Green PCR master mix (TransStart). Data were collected using Bio-Rad chromo 4 real-time PCR detector. All expressions were normalized against the *Ubiquitin* gene. The primers used are listed at Additional file 1: Table S1.

### Germination and abiotic stress tolerance assay

Dehulled seeds were surface-sterilized and planted on half-strength MS medium supplemented with indicated concentration of ABA (A1049, Sigma-Aldrich). Seed germination was defined as the coleoptiles emerged from the seed and scored every 12 h for 5 days. Three independent T3 homologous transgenic lines and the control Longjing 11 were used for stress tolerance experiments. For the dehydration treatment, rice plants grown for 3 weeks were withhold water for 10 days and then rehydrated and grown under normal conditions for 7 days. For cold treatment, 14-day-old seedlings were transferred to 10 °C for 4 days and then returned to normal growth conditions for 7 days. The survival rates were recorded. Approximately 50 seedlings of each line were used for each experiment, and three replicates of each experiment were performed. Tests for statistical analysis between transgenic lines and the controls were performed using Microsoft excel 2007.

### Water loss assay and measurement of relative electrolyte leakage

For water loss assay, leaves of control and *OsPYLs* transgenic plants grown under normal conditions were detached from 3-week-old seedlings and weighed immediately on a piece of weighing paper, and then placed on a laboratory table and weighed at indicated time intervals. Three replicates were performed for each line.

For relative water content measurement (RWC), Three-week-old seedlings of control and three independent OsPYLs transgenic lines were withhold water. The protocol was as described by (Barrs and Weatherley 1962). RWC was measured until the leaf cannot expand during dipped in the water. 

Two-week-old seedlings of control and three independent *OsPYLs* transgenic lines were transferred into a 10 °C chamber. At 0 d, 2 d and 3 d, 0.5 g of leaves were harvested from each of ten plants. Leaf fragments were immersed in 6 mL deionized water and shaken at 100 rpm at 25 °C for 2 h, and electrical conductivity was determined (C1). The samples were then boiled for 20 min, and the total conductivity was determined again (C2) after cooling to room temperature. Relative ion leakage (%) was calculated as C1/C2 × 100.

## Additional file

Additional file 1: Figure S1. Interaction of OsPYLs and OsPP2Cs in yeast two-hybrid. **Figure S2.** Pylogenetic analysis of PYLs family members in Rice and Arabidopsis. **Table S1.** Primers used in this study. (DOCX 8631 kb)
