# Supplementary material for: Characterization and Functional Analysis of Pyrabactin Resistance-Like Abscisic Acid Receptor Family in Rice
Source: Rice (N Y). 2015 Sep 11;8:28. doi: 10.1186/s12284-015-0061-6 (PMC4567572; doi:10.1186/s12284-015-0061-6)
Supplement: Additional file 1: Figure S1. — Interaction of OsPYLs and OsPP2Cs in yeast two-hybrid. Figure S2. Pylogenetic analysis of PYLs family members in Rice and Arabidopsis. Table S1. Primers used in this study. (DOCX 8631 kb) [file 12284_2015_61_MOESM1_ESM.docx]

**Supplementary data**

Figure S1. Interaction of OsPYLs and OsPP2Cs in yeast two-hybrid.


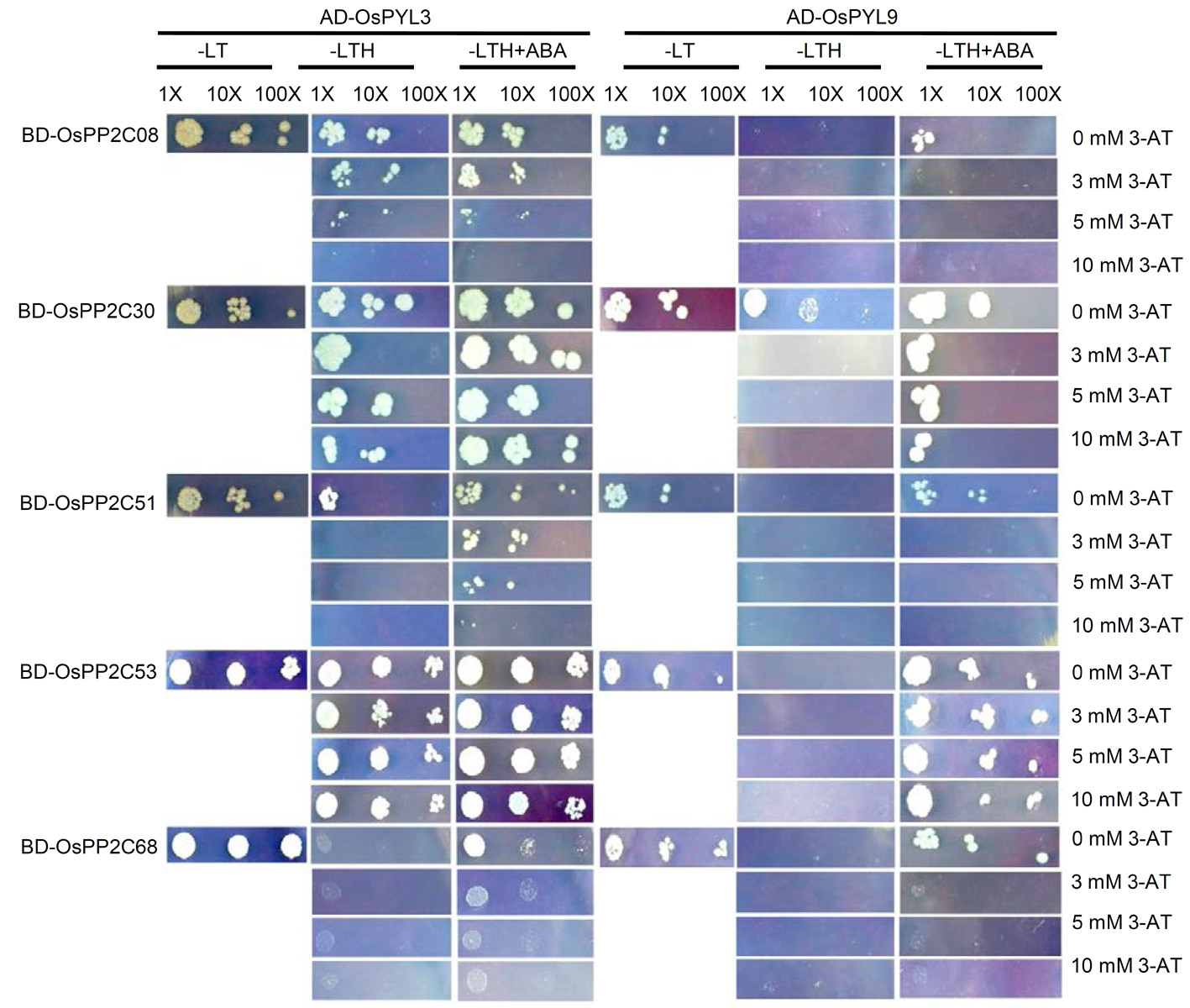


Using the OsPYL3 and OsPYL9 as example, the picture of interaction between OsPYL and OsPP2Cs was shown. The suspended cells containing corresponding OsPYL and OsPP2C constructs were spread on plates containing SD/-Leu/-Trp and SD/-His/-Leu/-Trp medium supplied with indicated concentration of 3-AT (3-amino-1, 2, 4-triazole) and ABA. The interactions were observed after 4 days of incubation at 30°C. The experiments were repeated three times with similar results.

Figure S2. Phylogenic tree of OsPYLs and AtPYLs

**
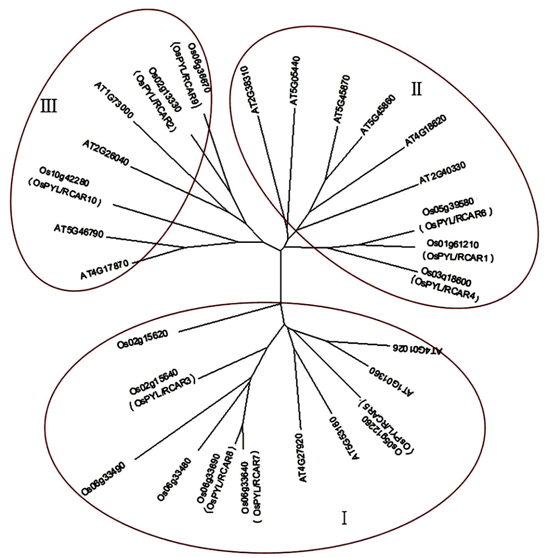
**

**Table S1**. Primers used for cloning, quantitative RT-PCR and RT-PCR

| Gene  name | Gene location | Usage | Forward (5’-3’) | Reverse (5’-3’) |
| --- | --- | --- | --- | --- |
| *OsPYL1* | Os01g61210 | *pENTR* | caccATGCCGTACGCCGCCGTACGTCC | TCATGCATGATCGATCGATCCG |
| *OsPYL2* | Os02g13330 | *pENTR* | caccATGGAGCCCCACATGGAGAGGGC | CTAATGGTGGTTGCCGGCGGCCGG |
| *OsPYL3* | Os02g15640 | *pENTR* | caccATGGTGGAGGTGGGAGGAGGAG | TCACCGGTCGAGGGGCTCGGTTTG |
| *OsPYL4* | Os03g18600 | *pENTR* | caccATGCCGTGCATCCCGGCGTCCAG | TCACGAGCCGGCGGCCCTCGCGC |
| *OsPYL5* | Os05g12260 | *pENTR* | caccATGGTGGGGCTTGTGGGAGG | CTACTGTTCAAGTGGCGAGGTGG |
| *OsPYL6* | Os05g39580 | *pENTR* | caccATGATGCCGTACACCGCTCCAC | CTAGGCGGCGCGCGGCGCGGCG |
| *OsPYL7* | Os06g33640 | *pENTR* | caccATGAACGGCGCTGGTGGTGCG | TCAAGGATTGGCAAGGCGCTCCT |
| *OsPYL8* | Os06g33690 | *pENTR* | caccATGAACGGCGTTGGTGGGGCGG | TCAAGGATTGGCAAGGCGCTCCT |
| *OsPYL9* | Os06g33670 | *pENTR* | caccATGGAGGCGCACGTGGAGAGGG | CTAGTCGCGCCGCCGCGAAGCAGA |
| *OsPYL10* | Os10g42280 | *pENTR* | caccATGGAGCAGCAGGAGGAAGTGC | CTATTCCGCCGCCGCCGGTGGAG |
| *OsPP2C08* | Os01g46760 | *pENTR* | ATGAGCAGTGACACAAGCAGGCG | CTACCTACCGTTCTCTTTGCTCG |
| *OsPP2C30* | Os03g16170 | *pENTR* | ATGGCGGAGATTTGCTGCGAGG | TCACAATTTCCGGCGCCTGAGA |
| *OsPP2C51* | Os05g49730 | *pENTR* | ATGAGGGAGACGGGCGCGACGGAT | TCAAGCTGCCCTGCTCTTGAG |
| *OsPP2C53* | Os05g51510 | *pENTR* | ATGGAGGACCTCGCCCTGCCC | TCATGCTTTGCTCTTGAACTTC |
| *OsPP2C68* | Os09g15670 | *pENTR* | ATGTCGATGGCGGAGGTGTGCTGT | CTACAAGGCGTTGCCTCGCCGG |
| *OsPYL1* | Os01g61210 | Q-PCR | CAGAGGAAAAAGAAGGCAACGAC | GCACCACGGTGGAGAAGCA |
| *OsPYL2* | Os02g13330 | Q-PCR | GCAGCGGTACAAGCACTTC | CGTCGAGGATCTCGAGGC |
| *OsPYL3* | Os02g15640 | Q-PCR | AAGGGAACATTGAGATTGGC | CGGTCAGGATGGAGGAGTAA |
| *OsPYL4* | Os03g18600 | Q-PCR | AGCCCCAGGCGTACAAGC | GTGGTGACCGAGAGGTAGTTC |
| *OsPYL5* | Os05g12260 | Q-PCR | CATCCTCAGCGTCAAGTTCG | TCACAAGCGTCCCTGGTCT |
| *OsPYL6* | Os05g39580 | Q-PCR | ATGATGCCGTACACCGCTCC | CGCCGCCTTCAACACTCC |
| *OsPYL7* | Os06g33640 | Q-PCR | GAGCAGCGGCAGGGAAGT | TTGGCGACGAACGAGGTG |
| *OsPYL8* | Os06g33690 | Q-PCR | CAGAGGAAAAAGAAGGCAACGAC | GCACCACGGTGGAGAAGCA |
| *OsPYL9* | Os06g33670 | Q-PCR | GGCGGAGGACACCAGGAT | CGACGATTTATTGACGAGG |
| *OsPYL10* | Os10g42280 | Q-PCR | CCACCACGACGACAACG | ATGGTGAAGCCGAAGACG |
| *LEA3* | Os05g46480 | Q-PCR | AGACCTCCAGCACGTCGCAG | GCCTGTTGGAGGACGCTGCC |
| *RAB16A* | Os11g26790 | Q-PCR | CAACGCTCCGGCAGCTCCAG | ATGCTGCTGCTCGCCCTTGT |
| *OsABA45* | Os12g29400 | Q-PCR | AGAGAGGGGACAGCCCGATG | AGGCTCAGCTTCCCCATCGC |
| *ubiquitin* | Os05g06770 | Q-PCR | ACCACTTCGACCGCCACTACT | ACGCCTAAGCCTGCTGGTT |
| *ubiquitin* | Os05g06770 | RT-PCR | CCTCGGACACCATCGACAACGTG | CGCCCCCAAAGAACAGGAGCCTA |
| *OsPYL9* | Os06g33670 | RT-PCR | GCAGCGGTACAAGCACTTC | CTAGTCGCGCCGCCGCGAAGCAGA |
